# Transcriptional profiling of skeletal muscle reveals starvation response and compensatory growth in *Spinibarbus hollandi*

**DOI:** 10.1186/s12864-019-6345-2

**Published:** 2019-12-05

**Authors:** Yang Yang, Huiqiang Zhou, Liping Hou, Ke Xing, Hu Shu

**Affiliations:** School of Life Science, Guangzhou University, Guangzhou, 510006 China

**Keywords:** *Spinibarbus hollandi*, Starvation response, Compensatory growth, Muscle

## Abstract

**Background:**

*Spinibarbus hollandi* is an economically important fish species in southern China. This fish is known to have nutritional and medicinal properties; however, its farming is limited by its slow growth rate. In the present study, we observed that a compensatory growth phenomenon could be induced by adequate refeeding following 7 days of fasting in *S. hollandi*. To understand the starvation response and compensatory growth mechanisms in this fish, the muscle transcriptomes of *S. hollandi* under control, fasting, and refeeding conditions were profiled using next-generation sequencing (NGS) techniques.

**Results:**

More than 4.45 × 10^8^ quality-filtered 150-base-pair Illumina reads were obtained from all nine muscle samples. De novo assemblies yielded a total of 156,735 unigenes, among which 142,918 (91.18%) could be annotated in at least one available database. After 7 days of fasting, 2422 differentially expressed genes were detected, including 1510 up-regulated genes and 912 down-regulated genes. Genes involved in fat, protein, and carbohydrate metabolism were significantly up-regulated, and genes associated with the cell cycle, DNA replication, and immune and cellular structures were inhibited during fasting. After refeeding, 84 up-regulated genes and 16 down-regulated genes were identified. Many genes encoding the components of myofibers were significantly up-regulated. Histological analysis of muscle verified the important role of muscle hypertrophy in compensatory growth.

**Conclusion:**

In the present work, we reported the transcriptome profiles of *S. hollandi* muscle under different conditions. During fasting, the genes involved in the mobilization of stored energy were up-regulated, while the genes associated with growth were down-regulated. After refeeding, muscle hypertrophy contributed to the recovery of growth. The results of this study may help to elucidate the mechanisms underlying the starvation response and compensatory growth.

## Background

Unfavorable environmental conditions, such as extreme temperature, depression, and hypoxic conditions, and human activities, such as transportation, can inhibit feeding behavior in fish, resulting in the starvation of fish in aquaculture. Starvation can influence growth, development, reproduction, and immunity in animals [1, 2]. Organisms usually maintain metabolic homeostasis during starvation by changing their behavior and activating various physiological and biochemical adaptive mechanisms. Recent studies have shown that lipid metabolism could be increased, and energy reserves could be mobilized, to cope with starvation in *Piaractus mesopotamicus* [3]. In rainbow trout (*Oncorhynchus mykiss*), it was found that genes related to protein degradation and amino acid metabolism increased, while genes involved in protein synthesis decreased under starvation [4]. In yellow croaker (*Larimichthys crocea*), a number of genes associated with carbohydrate metabolism also increased during fasting conditions [5]. In some fish species, food shortages have been shown to weaken immunity and inhibit body growth because of decreased energy consumption [6, 7].

Previous studies have shown that some fish have the ability to tolerate short-term food deprivation or fasting. Animals fed to satiation following continuous fasting have the potential to exhibit increased feeding intensity compared with continuously fed controls, resulting in the acceleration of growth; this phenomenon is defined as compensatory growth [8], which is known to occur in a wide range of fish species, such as Atlantic salmon (*Salmo salar*) [9], barramundi (*Lates calcarifer*) [10], channel catfish (*Ictalurus punctatus*) [11], tongue sole (*Cynoglossus semilaevis*) [12], European minnow (*Phoxinus phoxinus*) [13], and gibel carp (*Carassius auratus gibelio*) [14]. However, the regulatory mechanisms governing this phenomenon have not been thoroughly elucidated in fish.

Musculature provides the largest store of protein in the body of fish; muscle proteins are the most important energy source and are preferentially mobilized in vital organs during long-term food deprivation [15]. Furthermore, the muscle is the main edible tissue of most fish species and accounts for at least 50% of the body weight in most commercial fish species [16]. Previous studies have shown that muscle may play an important role in maintaining normal metabolism during dietary restriction due to an increase in protein degradation of the white muscle during starvation [15]. The investigation of the morphology and molecular changes in muscle during food deprivation can facilitate a better understanding of the starvation response and compensatory growth mechanisms.

*Spinibarbus hollandi* (also called army fish) is an endemic Cyprinidae species in southeastern China and is primarily distributed in Guangdong, Guangxi, Hunan, Hubei, Fujian, and Anhui Provinces. This fish is omnivorous, is easy to rear, and has already received increasing attention owing to its high nutritional and medicinal value [17]. However, the low growth rate seriously restricts the production efficiency and popularization of *S. hollandi* [17]. In addition, this fish species is sensitive to external stimuli, environmental changes and artificial stimulation, which can easily result in fasting for several days. In our previous work, we attempted to enhance the growth rate of *S. hollandi* by improving the rearing conditions and crossbreeding. The molecular markers associated with growth traits were developed in *S. hollandi* [17, 18]. However, studies on the effects of fasting and refeeding on the growing status of this species are deficient. The underlying physiological responses and gene expression changes associated with growth depression due to feed restriction have not been elucidated.

In the present study, the growth compensation phenomenon was observed in *S. hollandi.* To understand the regulatory mechanisms underlying the starvation response and growth compensation, comparative transcriptome analyses on skeletal muscle of different feeding statuses were performed in *S. hollandi*. The results showed that muscle overgrowth is an important reason for growth compensation. Therefore, a histological analysis of muscle was carried out to verify the conclusions obtained from transcriptome data.

## Results

**Fig. 1:**
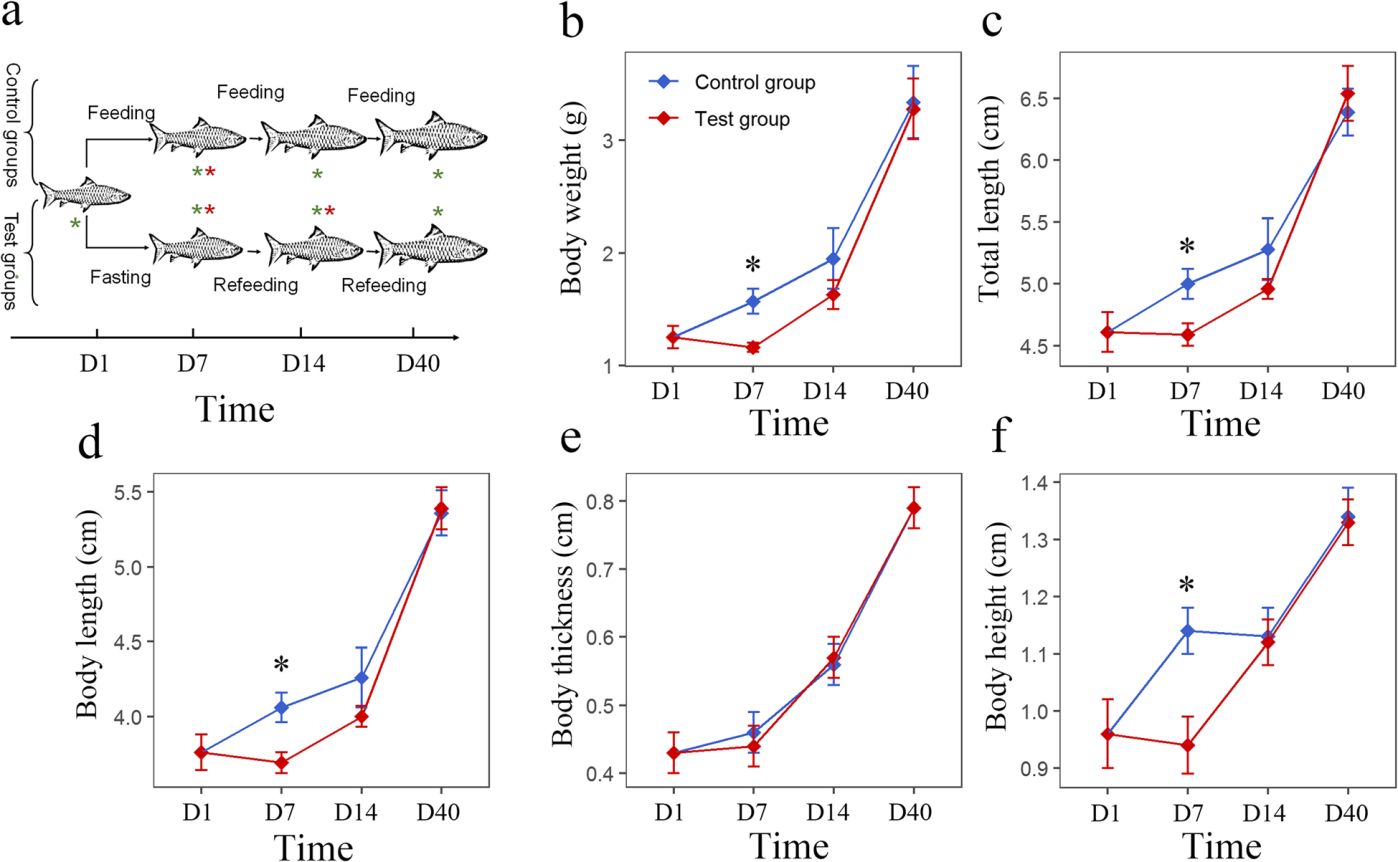
Experimental procedure, growth curves. **a** Illustration of the experiment and sample processes performed in this study. Red asterisks indicate that groups were used for RNA-seq; green asterisks indicate that groups were used for qRT-PCR; the horizontal axis indicates the time of experiment. **b–f** Growth curve of body weight, total length, body length, body thickness and body height, respectively. Asterisks indicate significant differences between the control and test groups (* *p* < 0.05; *Student’s t-test*)

As shown in Fig. 1a, a total of 210 mixed-sex, full-sib *S. hollandi* were randomly divided into a test group and a control group. For the test group, fish were first deprived of food for 7 days and then fed twice a day to apparent satiation for 33 days. For the control group, fish were fed twice a day continuously for 40 days (Fig. 1a). Both groups were sampled sequentially on days 0, 7, 14 and 40, and the growth traits, including weight, body length, total length, thickness and height, were measured. The muscle tissue transcriptomes were profiled by RNA-sequencing (RNA-seq) at day 7 for the control group and test group (after 7 days of fasting) and 14 for the test group (after 7 days of refeeding).

### Compensatory growth after fasting-refeeding treatment

After fasting, with the exception of body thickness, the body weight, total length, body length, and body height were decreased and significantly lower than those of the control group (*p* < 0.05 *Student’s t-test*; Fig. 1). After 7 days of refeeding, no remarkable difference could be detected between the test group and the control. Finally, at the end of the experiment, all growth traits were recovered (Fig. 1). Taken together, these results suggest effective growth compensation in *S. hollandi* after fasting-refeeding treatment. The daily gains of growth traits can be found in Additional file 1: Figure S1.

### Sequencing data and de novo assembly

The variation among individuals was minimized by mixing equal amounts of RNA from five samples in the same group. For each group, three replicated mixed RNA pools were generated and used for cDNA library preparation and RNA-seq thereafter. A total of 4.63 × 10^8^ raw reads (150 bp) were obtained from nine cDNA libraries. After removing the low-quality sequences, approximately 4.45 × 10^8^ clean reads with 66.71 Gb sequences were generated. Then, 355,268 transcripts (ranging from 201 to 27,096 bp) were generated through de novo assembly. The longest transcript of a gene was regarded as a unigene for further analyses. A total of 156,735 unigenes with 103.5 Mb sequences were obtained with an average length of 660 bp. The N50 and N90 lengths were 1061 and 224 bp, respectively.

### Annotation and functional analysis of muscle unigenes

Among all unigenes, 142,918 (91.18%) could be annotated in at least one database of non-redundant protein (Nr), non-redundant nucleotide (Nt) NCBI database, Pfam, KOG, Swiss-Prot, KEGG, and GO, with 10,438 (6.65%) unigenes annotated in all these databases. Approximately 54,408 (34.71%) unigenes were annotated in the Nr database, of which nearly 66.5% had the highest similarity with *Danio rerio* followed by *Astyanax mexicanus* (5.8%), *Clupea harengus* (2.5%), *Larimichthys crocea* (1.8%), and *Oncorhynchus mykiss* (1.7%).

**Fig. 2:**
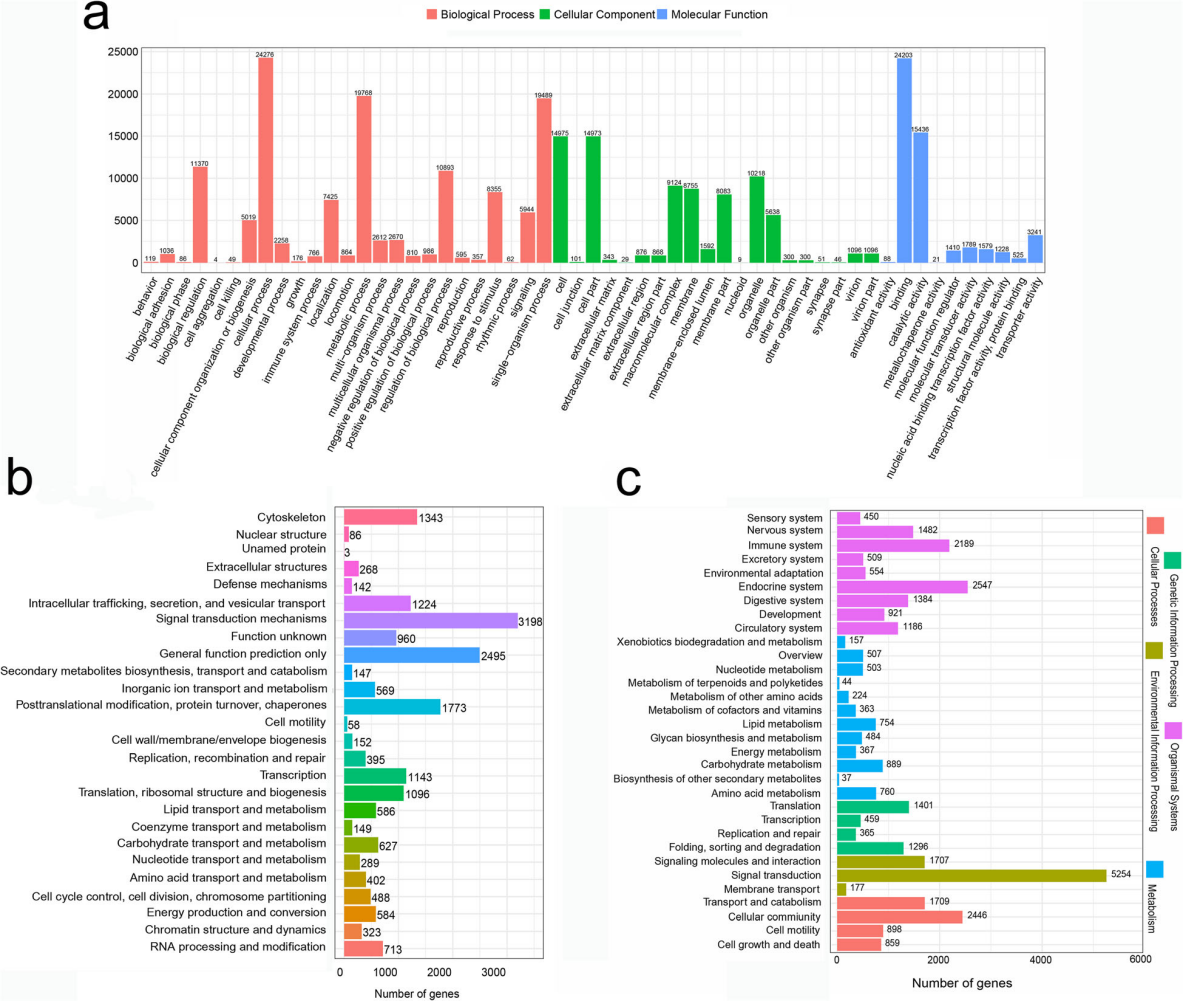
Annotation and function analysis of unigenes in *S. hollandi.* **a** Classification of the assembled unigenes in the Gene Ontology (GO) database; **b** Classification of the assembled unigenes in the Cluster of Orthologous Groups (COG) database; **c** Classification of the assembled unigenes in the Kyoto Encyclopedia of Genes and Genomes (KEGG) database

The functional prediction and classification of all unigenes were performed against the GO database. A total of 42,174 (26.91%) unigenes were classified into 55 GO terms, including 10 molecular function, 20 cellular components, and 25 biological process terms. Cellular process, binding, metabolic process, single-organism process, and catalytic activity were the five most abundant functional terms at the second GO level (Fig. 2a). Furthermore, the assembled unigenes were aligned to the COG database for phylogenetic classification. A total of 16,978 (10.83%) unigenes were divided into 26 functional categories (Fig. 2b); the largest category was signal transduction mechanisms with 3198 unigenes followed by general function prediction only (2495); posttranslational modification, protein turnover, chaperones (1773); and cytoskeleton (1343). The biological pathways were analyzed by annotating all assembled unigenes against the KEGG database, and 29,841 (19.04%) unigenes were matched in five categories, including organismal systems (11,222; 37.61%), environmental information processing (7138; 23.92%), cellular processes (5912; 19.81%), metabolism (5089; 17.05%), and genetic information processing (3521; 11.80%). These categories contained 232 pathways with 1 to 1147 unigenes that were involved in an individual pathway. Among all KEGG pathways, the PI3K-Akt signaling pathway, focal adhesion, regulation of actin cytoskeleton, endocytosis, and MAPK signaling pathway were significantly enriched with more than 800 unigenes (Fig. 2c).

### Identification of DEGs in different feeding conditions

**Fig. 3:**
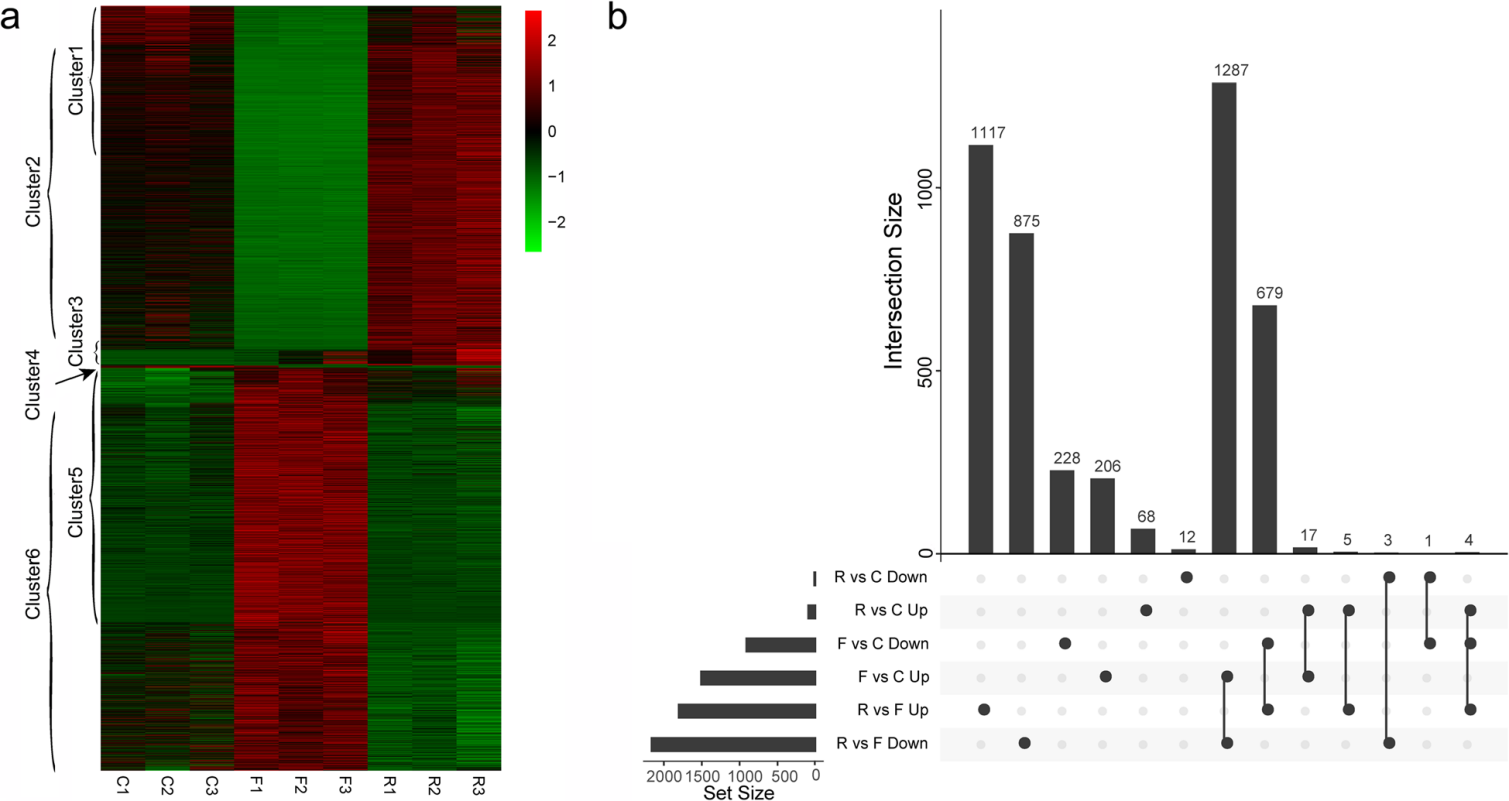
Overall transcriptome profiles of all DEGs. **a** Heatmap of all DEGs. Green and red indicate the low and high expression levels, respectively. C1, C2 and C3 indicate the control group, F1, F2 and F3 indicate the fasting group, and R1, R2 and R3 indicate the refeeding group. Cluster1 contains genes significantly down regulated in fasting group compared to control, with the exception of genes also significantly changed in refeeding group compared to control; Cluster2 contains genes significantly down regulated in fasting group compared to refeeding group, with the exception of genes also significantly changed in refeeding group compared to control; Cluster3 contains genes significantly up regulated in refeeding group compared to control; Cluster4 contains genes significantly down regulated in refeeding group compared to control; Cluster5 contains genes significantly up regulated in fasting group compared to control, with the exception of genes also significantly changed in refeeding group compared to control; Cluster6 contains genes significantly up regulated in fasting group compared to refeeding group, with the exception of genes significantly changed in refeeding group compared to control. The full list of these DEGs can be found in Additional file 2: Table S2. **b** UpSet chart showing intersection between different DEG sets from various comparisons. The vertical bar plot reports the intersection size, the dot plot reports the set participation in the intersection, and the horizontal bar plot reports the set sizes

A total of 4503 unigenes showed significant differential expression among the control, fasting and refeeding groups. Of these differentially expressed genes (DEGs), 2422 were found between the control and fasting groups, 110 were found between the control and refeeding groups, and 3970 were found between the fasting and refeeding groups (Fig. 3a, b and Additional file 2: Table S2). The overall expression profiles of control group and refeeding group were similar *(R*^*2*^ = 0.95, *p* < 2.2e-16; only the 4503 DEGs were considered), and the DEGs identified from fasting compared to control comparison and fasting compared refeeding comparison were largely overlapped (Fig. 3a, b). We mainly focused on genes significantly changed in fasting group compared to control and genes significantly changed in refeeding group compared to control in our following analysis.

### Analysis of DEGs after fasting in *S. hollandi*

**Fig. 4:**
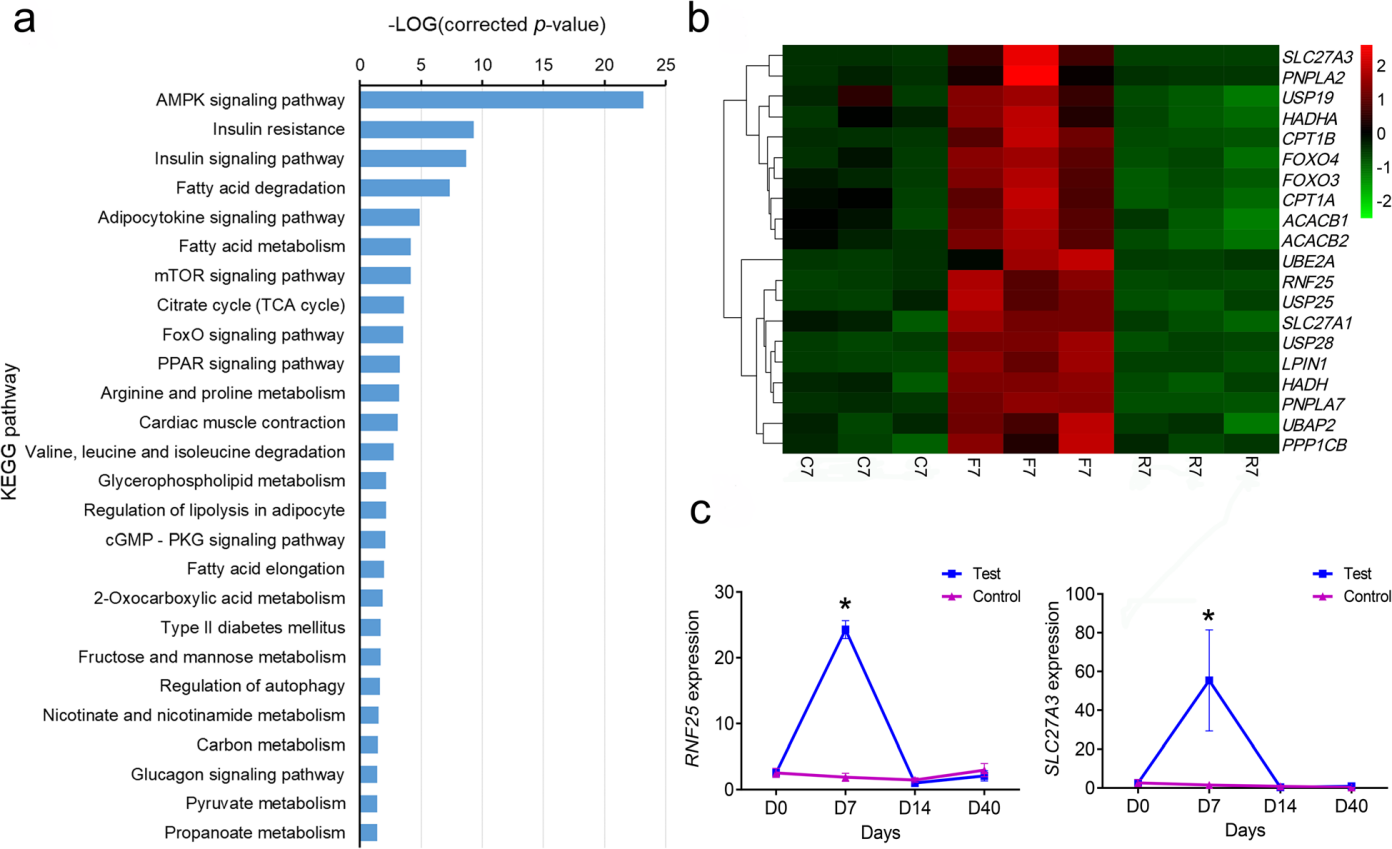
Analysis of up-regulated differentially expressed genes (DEGs) during fasting and refeeding. **a** classification of the up-regulated DEGs in the Kyoto Encyclopedia of Genes and Genomes (KEGG) database; **b** expression profiles of the parts of up-regulated DEGs from the RNA-seq result; **c** *RNF25* and *SCL27A3* expression profiles in the control and test groups from the qRT-PCR result. Asterisks indicate significant differences between the control and test groups (* *p* < 0.05; *Student’s t-test*)

After 7 days of fasting, we detected 2422 DEGs, which included 1510 up-regulated and 912 down-regulated genes (Additional file 2: Table S2). Approximately 331 up-regulated DEGs mapped to 284 KEGG pathways, 26 of which were significantly enriched (corrected *p-value* < 0.05; Fig. 4a). The top five significantly enriched pathways were the AMPK signaling pathway (ko04152), insulin resistance (ko04931), insulin signaling pathway (ko04910), fatty acid degradation (ko00071), and adipocytokine signaling pathway (ko04920). Many metabolism-relevant genes were significantly up-regulated in the muscle after 7 days of fasting (Fig. 4b). The genes associated with lipid metabolism (*PNPLA2*, *SLC27A1*, *SLC27A3*, *HADH*, *HADHA*, *LPIN1*, *ACACB1*, *ACACB2*, *CPT1A*, and *CPT1B*), protein degradation and metabolism (*USP19*, *RNF25*, *USP25*, *USP28*, *UBE2A*, *PSMB7*, and *PSMG3*), and glycometabolism (*PPP1CB*, *PFKFB1*, *GSK3B*, and *GYS*) were significantly up-regulated. In addition, some genes associated with signal transduction (*FOXO3*, *FOXO4*, *PIK3*, and *ARS1*) were up-regulated during fasting. To validate the results from RNA-seq, the expression profiles of *RNF25* and *SLC27A3* were analyzed by using quantitative PCR (qPCR). The results were similar to the RNA-seq results, in which the expression of these two genes was significantly increased after 7 days of fasting and then returned to normal levels after refeeding (Fig. 4c).

**Fig. 5:**
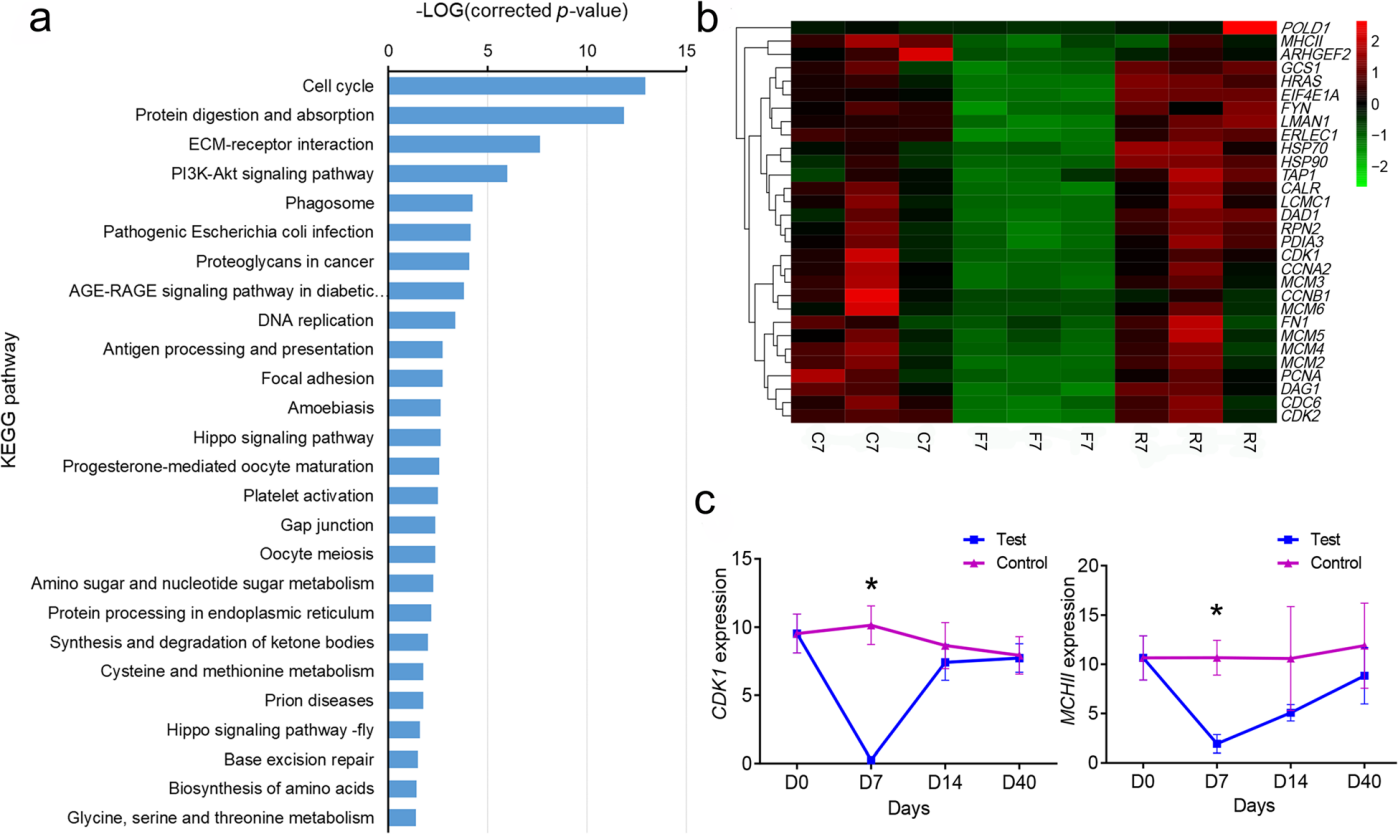
Analysis of down-regulated differentially expressed genes (DEGs) during fasting and refeeding. **a** classification of the down-regulated DEGs in the Kyoto Encyclopedia of Genes and Genomes (KEGG) database; **b** expression profiles of some down-regulated DEGs from the RNA-seq result; **c** the expression of *CDK1* and *MHCII* in the control and test groups from the qRT-PCR result. Asterisks indicate significant differences between the control and test groups (* *p* < 0.05; *Student’s t-test*)

A total of 316 down-regulated DEGs were mapped to 243 pathways in the KEGG database, of which 26 pathways (Fig. 5a) were significantly enriched (corrected *p*-value < 0.05). The top five significantly enriched pathways were cell cycle (ko04110), protein digestion and absorption (ko04974), ECM-receptor interaction (ko04512), PI3K-Akt signaling pathway (ko04151), and phagosome (ko04145). The expression profiles of some of the down-regulated DEGs from RNA-seq are shown in Fig. 5b. Genes associated with the cell cycle (*HRAS*, *CDK1*, *CDK2*, *CDC6*, *FGF6*, *CCNA2*, *Wee1*, and *CCNB1*), DNA replication (*EIF4E1A*, *PCNA*, *POLD1*, *MCM1*, *MCM2*, *MCM3*, *MCM4*, *MCM5*, and *MCM6*), extracellular matrix (*ITGA4*, *COL1A*, *LAMC1*, *FN1*, *TN*, and *DAG1*), protein synthesis (*RPN2*, *GCS1*, *HSPA5*, *PDIA3*, *PDIA4*, *LMAN1*, and *ERLEC1*), and immune and stress tolerance (*ARHGEF2*, *TUBA*, *TUBB*, *FYN*, *MHCII*, *TAP1, HSP70, HSP90*, and *CTSB*) were significantly down-regulated. The expression of *CDK1* and *MHCII* in the control and test groups was analyzed using qRT-PCR (Fig. 5c). The expression of *CDK1* was significantly down-regulated after 7 days of fasting, and then returned to normal levels after refeeding. *MHCII* was significantly down-regulated after 7 days of fasting, and its expression gradually increased.

### Analysis of DEGs after refeeding

After refeeding, we detected a total of 3970 DEGs between the fasting and refeeding groups, of which 1805 were up-regulated and 2165 were down-regulated (Additional file 2: Table S2). Compared with the control group, a total of 110 genes were significantly changed, including 94 DEGs and 16 DEGs that were up-regulated and down-regulated. It’s worth noting that among the 94 up regulated genes in the refeeding group, 38 of them increased in one or two fasting samples too. These genes, thus, may not related to the compensatory growth. In the remaining DEGs, 9 genes involved in myofiber structure (*ACTC1*, *ACT2*, *ACTA1*, *MHC*, *MLC1*, *MYL2*, *TM1*, *TNT*, and *TNNT3*) were significantly up-regulated (Additional file 1: Figure S2 and Table 1). In addition, genes involved in collagen (*COL3A1*, *COL1A2*, and *COL5A2*), ribosomal protein (*RPL22L1*), protein synthesis (*INFB*), and energy metabolism (*ATPD*, *GAPDH*, *ND2*, and *PDHB*) were also up-regulated (Table 1).

**Table 1:** Up-regulated differentially expressed genes (DEGs) after refeeding

Gene_ID	log2FC	*padj*	Swissprot Description	Symbol
TRINITY_DN53185_c1_g5	14.71	5.04E-14	Uncharacterized protein ORF91	
TRINITY_DN28038_c0_g1	11.91	2.30E-10	Cell division protein FtsZ	FTSZ
TRINITY_DN53185_c1_g3	11.67	1.31E-12	Uncharacterized protein ORF91	
TRINITY_DN40074_c2_g1	11.21	1.49E-08	Sarcoplasmic calcium-binding protein 1	
TRINITY_DN51715_c1_g6	10.96	6.10E-11	Oligopeptide transport ATP-binding protein OppD	OPPD
TRINITY_DN46704_c4_g3	10.64	5.38E-07	Oligopeptide transport ATP-binding protein OppD	OPPD
TRINITY_DN39439_c1_g1	10.60	4.89E-08	Myosin heavy chain, muscle	MHC
TRINITY_DN51457_c0_g4	10.56	9.76E-06	Arginine kinase	
TRINITY_DN44733_c0_g1	10.38	7.90E-08	Myosin light chain alkali	MLC1
TRINITY_DN36404_c0_g1	10.34	5.91E-11	Collagen alpha-1(III) chain	COL3A1
TRINITY_DN36624_c0_g1	10.28	8.79E-07	Myosin regulatory light chain 2	
TRINITY_DN62802_c0_g1	10.10	2.75E-05	Ribosomal RNA small subunit methyltransferase H	RSMH
TRINITY_DN46916_c1_g3	9.53	2.78E-07	Tropomyosin	TM1
TRINITY_DN29972_c1_g3	9.50	4.91E-05	ATP synthase subunit beta	ATPD
TRINITY_DN75810_c0_g1	9.44	1.72E-03	DNA-directed RNA polymerase subunit beta’	RPOC
TRINITY_DN46916_c1_g2	9.26	1.53E-09	Troponin I	
TRINITY_DN51457_c0_g2	9.13	2.81E-02	Cytochrome c oxidase subunit 1	COI
TRINITY_DN29188_c0_g1	9.09	1.09E-06	Putative transposase InsK for insertion sequence element IS150	INSK
TRINITY_DN30901_c0_g1	8.90	1.08E-04	Membrane protein insertase YidC	YIDC
TRINITY_DN14841_c0_g1	8.88	4.80E-03	L-arabinose transport ATP-binding protein AraG	ARAG
TRINITY_DN51715_c1_g8	8.76	1.02E-03	Oligopeptide transport ATP-binding protein OppD	OPPD
TRINITY_DN55417_c1_g2	8.73	5.18E-03	Craniofacial development protein 2	CFDP2
TRINITY_DN6518_c0_g2	8.67	6.49E-04	tRNA-specific 2-thiouridylase MnmA	MNMA
TRINITY_DN54848_c1_g1	8.58	1.22E-02	Cingulin	CGN
TRINITY_DN41914_c0_g2	8.55	8.58E-04	von Willebrand factor	VWF
TRINITY_DN33939_c0_g1	8.53	1.43E-07	Variant surface antigen C	VLPC
TRINITY_DN53727_c0_g1	8.40	2.78E-07	Troponin T	TNT
TRINITY_DN42223_c1_g1	8.36	4.80E-07	Actin, alpha cardiac muscle 1	ACTC1
TRINITY_DN26420_c0_g1	8.06	1.21E-03	Fructose-bisphosphate aldolase	ALD
TRINITY_DN27809_c1_g2	8.00	2.00E-02	50S ribosomal protein L6	RPLF
TRINITY_DN50962_c2_g1	7.95	7.78E-03	Cytochrome c oxidase subunit 3	COIII
TRINITY_DN50547_c1_g2	7.92	4.03E-02	Collagen alpha-2(I) chain	COL1A2
TRINITY_DN29972_c1_g4	7.81	9.29E-03	Elongation factor Tu	TUF
TRINITY_DN21598_c0_g2	7.80	2.43E-02	Chaperone protein DnaK	DNAK
TRINITY_DN14984_c0_g1	7.79	2.54E-02	Pyruvate dehydrogenase E1 component subunit beta	PDHB
TRINITY_DN4314_c0_g1	7.78	3.33E-02	Putative permease MJ0326	MJ0326
TRINITY_DN71455_c0_g1	7.73	2.15E-04	Membrane-associated lipoprotein	UU045
TRINITY_DN27456_c0_g2	7.69	1.04E-03	Troponin C, isoform 1	
TRINITY_DN49873_c2_g2	7.67	2.37E-03	Cytochrome c oxidase subunit 1	MT-CO1
TRINITY_DN57252_c0_g1	7.65	8.30E-05	Putative ABC transporter ATP-binding protein MG187 homolog	MPN_134
TRINITY_DN76701_c0_g1	7.60	5.18E-03	Pyruvate dehydrogenase E1 component subunit beta	PDHB
TRINITY_DN9860_c0_g1	7.57	2.95E-04	Ascorbate-specific permease IIC component UlaA	ULAA
TRINITY_DN72099_c0_g1	7.43	1.14E-03	Lon protease	LON
TRINITY_DN37203_c0_g1	7.42	1.02E-03	Uncharacterized protein ORF91	
TRINITY_DN50867_c4_g2	7.39	3.41E-02	Collagen alpha-2(V) chain	COL5A2
TRINITY_DN39233_c1_g2	7.17	6.64E-03	Putative uncharacterized transposon-derived protein F52C9.6	F52C9.6
TRINITY_DN26948_c0_g1	7.15	6.88E-03	Troponin C, isotype gamma	
TRINITY_DN57481_c0_g1	7.12	8.41E-03	30S ribosomal protein S2	RPSB
TRINITY_DN42492_c1_g7	7.12	7.34E-03	Glyceraldehyde-3-phosphate dehydrogenase	
TRINITY_DN76302_c0_g1	7.09	1.22E-02	Uncharacterized protein PF11_0207	PF11_0207
TRINITY_DN20261_c0_g1	6.98	1.59E-02	NADH-ubiquinone oxidoreductase chain 2	MT:ND2
TRINITY_DN31759_c0_g1	6.96	4.28E-02	Translation initiation factor IF-2	INFB
TRINITY_DN38000_c0_g1	6.92	2.90E-02	RNA-directed DNA polymerase from mobile element jockey	POL
TRINITY_DN66334_c0_g1	6.80	4.92E-02	PTS system glucose-specific EIICBA component	PTSG
TRINITY_DN50552_c0_g2	3.93	2.48E-04	Collagen alpha-2(I) chain	COL1A2
TRINITY_DN41454_c2_g1	3.02	2.00E-02	Actin, alpha skeletal muscle	ACTA1
TRINITY_DN39818_c0_g2	2.52	4.02E-02	Actin, alpha skeletal muscle 2	ACT2
TRINITY_DN37767_c0_g1	2.23	2.36E-02	Thymosin beta	TMSB
TRINITY_DN48071_c0_g2	1.99	3.45E-03	60S ribosomal protein L22-like 1	RPL22L1
TRINITY_DN51452_c2_g2	1.98	7.99E-03	Troponin T, fast skeletal muscle	TNNT3
TRINITY_DN50109_c0_g2	1.94	4.60E-02	Endothelial lipase	LIPG

*P*-value <0.05 indicated different expression between control and fasting-refeeding groups

**Fig. 6:**
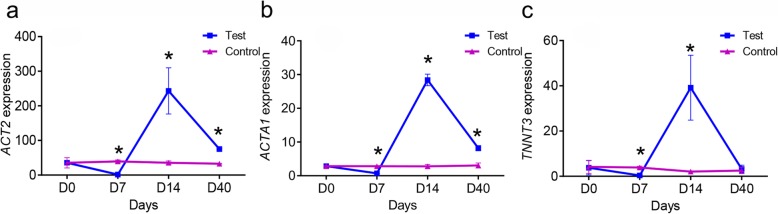
Expression profiles of some of the genes associated with myofibers under prolonged refeeding. **a**, **b** and **c** indicated the expression profiles of *ACT2*, *ACTA1* and *TNNT3*, respectively; Asterisks indicate significant differences between the control and test groups (* *p* < 0.05; *Student’s t-test*)

Three genes, *ACT2*, *ACTA1*, and *TNNT3*, were used for expression trend analysis between the control and test groups. The *ACT2* expression significantly decreased after fasting for 7 days (*p* = 0.013; *Student’s t-test, df = 4*) compared with the control group at the same point and then sharply increased and became significantly higher than that in the control group with refeeding for 7 days; after refeeding for 33 days, the *ACT2* expression decreased, but it was still significantly higher than that in the control group (*p* = 1.0 × 10^− 5^; *Student’s t-test, df = 4*) (Fig. 6a). The expression profile of *ACTA1* had the same pattern as that of *ACT2*: after fasting, its expression significantly decreased, whereas after refeeding, its expression significantly increased and then gradually decreased (*p* = 8.05 × 10^− 4^; *Student’s t-test, df = 4*) (Fig. 6b). The expression change of *TNNT3* was similar to *ACTA1* and *ACT2*; however, after refeeding for 33 days, no significant difference was noted in its expression between the control and refeeding groups (Fig. 6c).

### qRT-PCR validation of DEGs from RNA-Seq

**Fig. 7:**
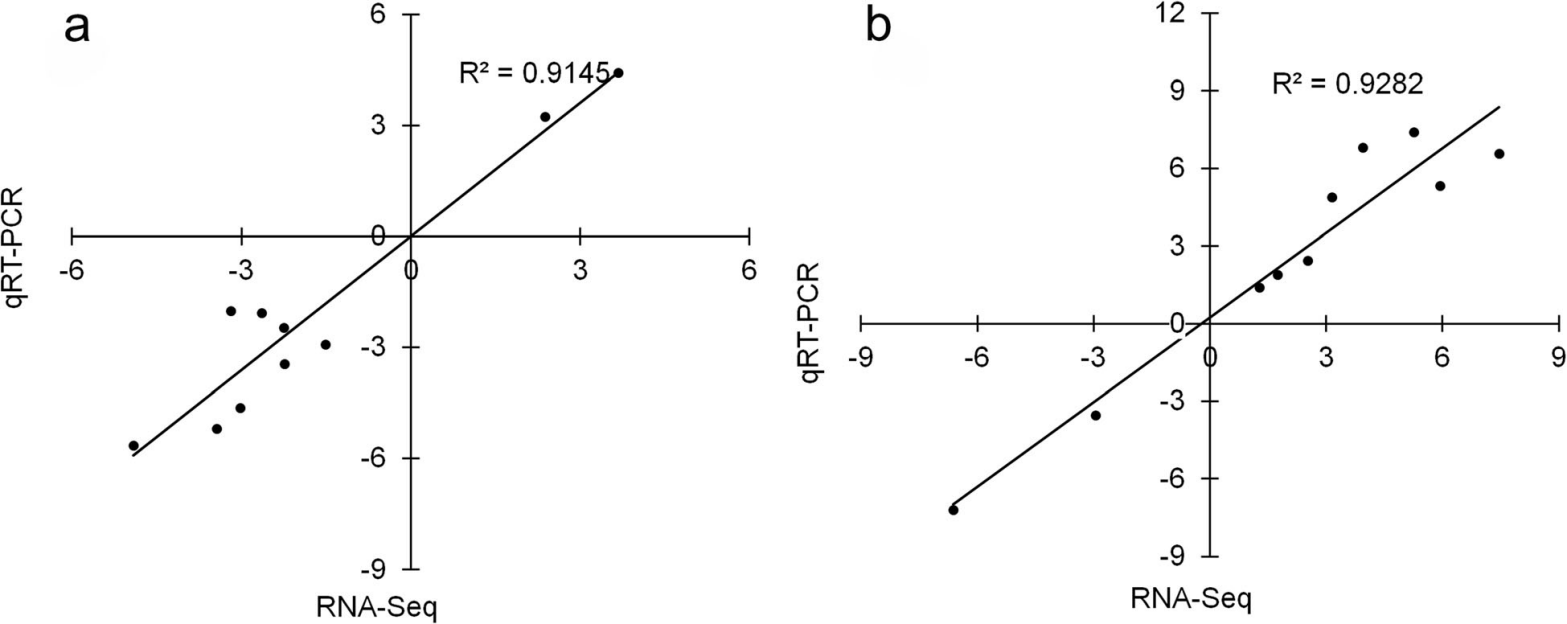
Correlation between the results of RNA-seq and qRT-PCR. **a** and **b** represent the correlations between the control and test group (after fasting) and between the control and test group (after refeeding), respectively. The X-axis numbers represent the log_2_ (fold-change) values from the RNA-seq results. The Y-axis numbers represent the log_2_ (fold-change) values from the qRT-PCR results

A total of ten DEGs of different functions were randomly selected to verify the RNA-seq results using quantitative real-time PCR (qRT-PCR), including genes involved in protein degradation (*RNF25*), lipid metabolism (*SLC27A3*), cell cycle (*CDK1* and *TMSB*), immunity (*MCHII* and *TAP1*), DNA replication (*MCM4*) and myofiber structure (*ACTA1, ACT2* and *TNNT3*). The expression changes of these genes were analyzed under fasting and refeeding conditions. Under both fasting and refeeding conditions, the gene expression changes obtained from qRT-PCR were remarkably consistent with those obtained from the RNA-Seq result, with *R*^*2*^ values of 0.9145 and 0.9282 (Fig. 7), respectively. The analyses confirmed the reliability and accuracy of the RNA-seq result.

### Changes in myofibers during fasting and refeeding periods

**Fig. 8:**
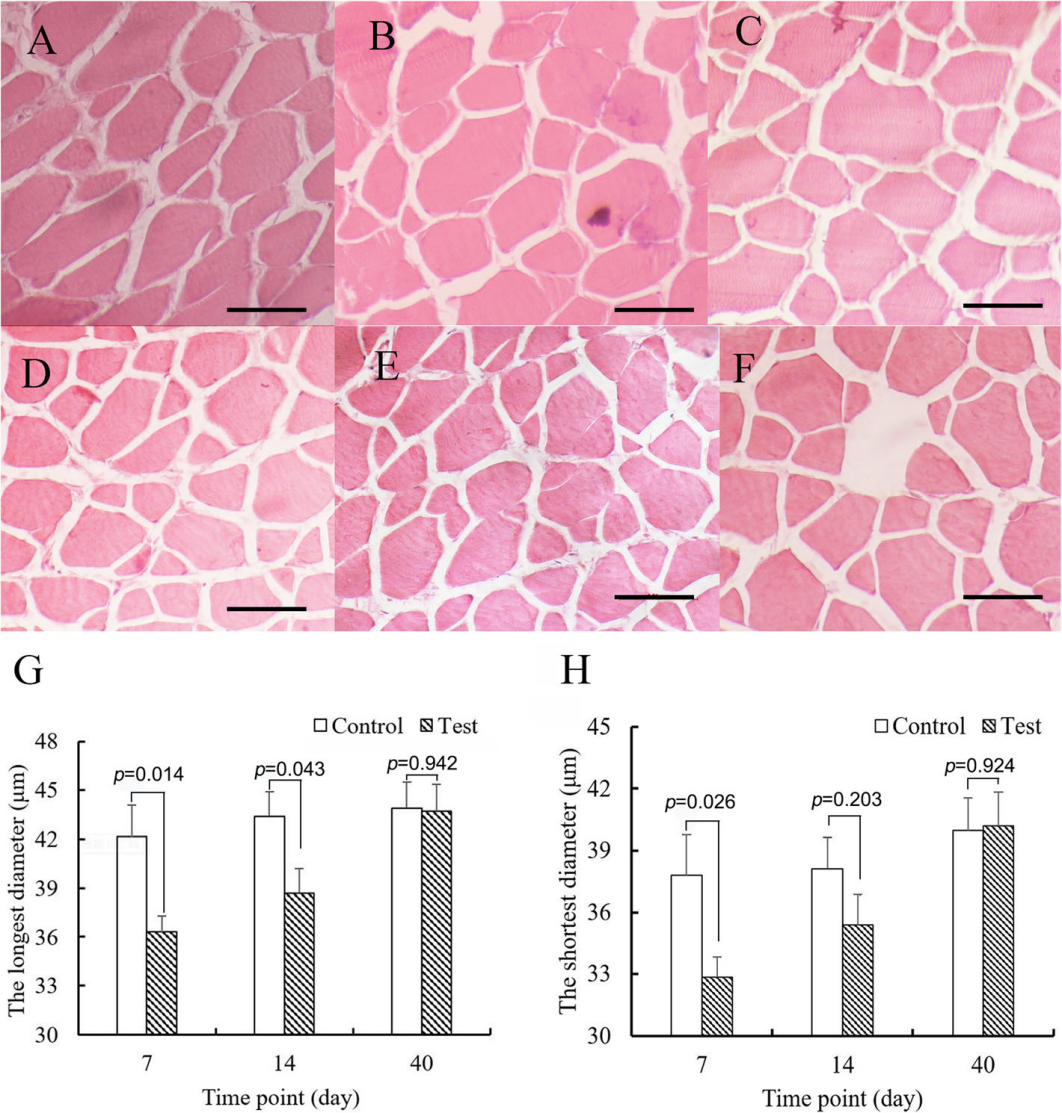
Changes in myofibers (**a-f**) indicate myofiber cross-sections in the control group at days 7, 14, 40 and the test group at day 7 (after 7 days fasting), 14 (after 7 days refeeding), 40 (after 33 days refeeding), respectively; **g** and **h** indicate the longest and shortest diameters of cross-section in the control and test groups, respectively. Asterisks indicate significant differences between the control and test groups (* *p* < 0.05; *Student’s t-test*; *n* = 5); bars = 50 μm

The dimensions of myofibers were analyzed using histology. The myofiber sections of the control groups are shown in Fig. 8a–c and those of the test groups are shown in Fig. 8d–f. The shortest and longest diameters of myofibers were measured (Fig. 8g, h). After 7 days, the shortest and longest diameters of the test group were 32.86 ± 0.98 and 36.30 ± 1.07 μm, respectively, which were significantly shorter than those of the control group (37.80 ± 1.96 and 42.14 ± 2.09 μm, *p* = 0.014 and *p* = 0.026; *Student’s t-test*). After 7 days of refeeding, the mean longest diameter of the test group of the refeeding group was still significantly shorter than that of the control group (*p* = 0.043; *Student’s t-test*); however, no significant difference was noted between their shortest diameters (*p* = 0.203; *Student’s t-test*). In addition, at the end of 40 days, no significant differences were noted in both diameters (*p* = 0.942 and *p* = 0.924, *Student’s t-test*).

## Discussion

In this study, a compensatory growth phenomenon was observed in *S. hollandi* that were refed after fasting for 7 days. During fasting, the growth of *S. hollandi* was inhibited; after 33 days of refeeding, the weight of the fasted fish almost recovered to the normal weight of the control group. In *S. hollandi*, starvation increased the expression of genes involved in fat, protein, and carbohydrate metabolism but reduced the expression of genes associated with the cell cycle, DNA replication, and immune and cellular structures. The compensatory growth seemed to mainly result from muscle overgrowth; many genes involved in myofiber development were significantly upregulated. Histological analyses of muscle suggested that starvation inhibited myofiber growth but that refeeding after fasting induced myofibril overgrowth. Fasting also inhibited the growth of *S. hollandi* overall; the body weight of fasted fish even decreased slightly during fasting. A reduction in body mass may be the most obvious response to starvation.

The body weight reduction after fasting has been reported in several fish species, including *Morone saxatilis* [19], *Stizostedion vitreum* [20], *Cynoglossus semilaevis* [12], and *Oncorhynchus mykiss* [21]. Refeeding could accelerate the growth of *S. hollandi* after a period of fasting; this phenomenon is known as compensatory growth and might be affected by feed intake and food utilization efficiency [22, 23]. This has been reported in many fish species, such as *Ctenopharyngodon idella* [24], *Oncorhynchus mykiss* [21], *Rutilus caspicus* [25], and *Megalobrama amblycephala* [26]. Although starvation and compensatory growth commonly exist in natural and production environments, the molecular mechanisms regulating the growth rates of skeletal muscle under both fasting and refeeding conditions have not been elucidated. The next-generation sequencing technology revolutionized the field of genomics; RNA-Seq can detect the transcriptome of the non-model species without known genomic information [27].

Through comparative transcriptome analysis, most of the up-regulated DEGs under starvation were involved in lipid metabolism, protein degradation and metabolism, and glycometabolism. *PNPLA2* is known to be important for triglyceride hydrolysis, and its up-regulation might enhance the hydrolysis of triglycerides and produce free fatty acids for other tissues in response to starvation [28]. *SLC27A* (*SLC27A1* and *SLC27A3*) is associated with fatty acid transport [29], and an increase in these genes may contribute to the mobilization of fat. *HADH* encodes an enzyme called 3-hydroxyacyl-CoA dehydrogenase, which is important for the beta-oxidation of short chain fatty acids in the mitochondria [30]; its increase may contribute to mobilizing the stored fatty acids to provide the necessary energy for the activity of organisms. CPT1s participate in the movement of acylcarnitine from the cytosol into the intermembrane of mitochondria [31], and its up-regulation contributes to fatty acid oxidation. *ACACBs* (*ACACB1* and *ACACB2*) serve as regulators of mitochondrial fatty acid oxidation through the inhibition of carnitine palmitoyltransferase 1 by its product malonyl-CoA [32], which reduces the fatty acid oxidation rate of organisms. Lipin-1 has phosphatidate phosphatase activity and plays a pivotal role in the maintenance of adipocytes [33]*.* The up-regulation of these genes may prevent excessive loss of fat during starvation conditions. During fasting, several up-regulated DEGs (*USP19*, *RNF25*, *USP25*, *USP28*, *UBE2A*, *PSMB7*, and *PSMG3*) are involved in the ubiquitin-proteasome system, which is one of the major protein degradation systems [34]. In addition, many genes were significantly enriched in amino acid catabolism-related KEGG pathways, including arginine and proline metabolism (ko00330), and valine, leucine, and isoleucine degradation (ko00280). The up-regulation of these genes indicated that fasting could accelerate the consumption of proteins and amino acids. *PPP1CB* encodes one of the three catalytic subunits of protein phosphatase 1, which is a serine/threonine-specific protein phosphatase known to be involved in glycogen metabolism, lipid metabolism, replication, pre-mRNA splicing and protein synthesis [35]. However, the direct connection between this gene and starvation response has not been determined. PFKFB1 is associated with the regulation of the synthesis and degradation of fructose-2,6-biphosphate, which is a rate-limiting enzyme in glucose homeostasis. This protein activates the glycolysis pathway and inhibits the gluconeogenesis pathway to produce more energy for organisms [36], its up-regulation may contribute to the regulation of glucose synthesis and metabolism. GSK3B and GYS are pivotal enzymes in glycogen metabolism [37, 38], which may be essential for the response to fasting in *S. hollandi*. However, the function of the two up-regulated genes is opposite, possibly because glycogen may be a buffer of glucose; when excessive stored energy is transformed into carbohydrates, glucose may be transformed into glycogen for temporary storage; when glucose concentration decreases, glycogen may be converted back into glucose. Up-regulated DEGs related to lipid metabolism, protein degradation and metabolism, and glycometabolism suggested that starvation increased the capacity to catabolize fats, proteins, and carbohydrates in *S. hollandi*. Similar results were found in other fish species: the genes related to fat and carbohydrate catabolism were up-regulated during fasting in large yellow croaker [7], and the genes associated with catabolic pathways and beta oxidation of fatty acids were significantly up-regulated in the muscle of rainbow trout after fasting for 1 month [4].

In addition to the up-regulated DEGs, a total of 912 genes were down-regulated by fasting. Most of these genes were involved in the cell cycle, DNA replication, extracellular matrix, protein synthesis, and immune and stimulus responses. A mitotic cell cycle is a complex process that is regulated by many factors. Cyclin (CCNA2 and CCNB1) can regulate cell cycle progress by interacting with cyclin-dependent kinases (CDK1 and CDK2). Wee1 is a nuclear kinase that can prevent cells from undergoing mitosis by inhibiting CDK1; it plays a critical role in the maintenance of normal mitosis during cell division [39, 40]. HRAS is a GTPase involved in the regulation of cell division [41]. F6F is a fibroblast growth factor (FGF) family member that possesses broad mitogenic and cell survival activities and is known to regulate muscle regeneration or differentiation [42]. DNA replication is a vital event in the mitotic cell cycle and showed signs of down-regulation by fasting. A mini-chromosome maintenance (MCM) complex formed by MCM proteins with DNA helicase activity might be involved in the formation of replication forks and recruitment of other DNA replication-related proteins [43]. PCNA is a cofactor of DNA polymerase delta, which acts as a scaffold in recruiting proteins involved in DNA replication [44]. *POLD1* encodes the catalytic subunit of the DNA polymerase delta complex [45], and CDC6 is an essential regulator of DNA replication and plays important roles in the transition from the S phase to the M phase of cell cycle progression [46]. Similar to our findings in *S. hollandi*, several genes associated with cell cycle and DNA replication were shown to be significantly down-regulated in *O. mykiss* during fasting [4, 21]. The extracellular matrix comprises a complex mixture of structural and functional macromolecules and is associated with many cellular processes, including growth, differentiation, morphogenesis, survival, and homeostasis [47]. The genes related to the extracellular matrix (*ITGA4, COL1A, LAMC1, FN1, TN*, and *DAG1*) were ulated and might reduce the growth and differentiation of myocytes during the fasting period; in addition, several genes associated with the cytoskeleton were down-regulated. Protein synthesis is essential for cellular processes. Genes associated with protein synthesis and modification pathways were down-regulated under fasting conditions, which might hinder the growth of *S. hollandi*. Genes associated with immune and stress tolerance were also down-regulated; heat shock proteins (HSPs) belong to the molecular chaperone family and play important roles in protein folding, as well as in protecting cells from stress. FYN is associated with T-cell signaling [48]; CTSB is a lysosomal cysteine protease associated with immunity [49]. TAP1 and MHCII are related to specific immunity [50] and are important parts of the immune system. The down-regulation of these DEGs suggested that fasting could weaken immunity and might increase morbidity in *S. hollandi* when threatened by pathogenic microorganisms. Previous studies have also shown that many genes related to immune and antimicrobial peptides were down-regulated in the liver following starvation in Atlantic salmon (*Salmo salar*) [51].

After refeeding, many genes associated with the structure of myofibers were up-regulated. Their expression levels were significantly higher than those in the control group. The sarcomere primarily consists of actin, myosin, tropomyosin, and troponin [52]; the overexpression of these proteins may promote muscle growth [53–55]. Collagens are major components of the perimysium and are essential for the stabilization of the extracellular matrix of muscle [56]. In trout, it was also observed that genes associated with myofiber and muscle remodeling were up-regulated 7 to 36 days post-refeeding [4], and the same research team suggested that compensatory muscle growth response resulted from the stimulation of hypertrophy [21]. Furthermore, several genes associated with energy metabolism (*ATPD*, *GAPDH*, *ND2*, and *PDHB*) were up-regulated; they might be required for meeting the energy requirement for muscle growth. In general, these findings indicated that the accelerated biosynthesis of myofibers resulted in compensatory growth following refeeding in *S. hollandi*. In histology analysis, the significant difference in the dimensions of myofibers showed that starvation can inhibit the growth of myofibers, which was in accord with results obtained from transcriptome analysis. After refeeding, the overgrowth of myofibers was consistent with the up-regulated expression levels of genes associated with muscle structure. Based on the above results, we suggest that muscle overgrowth may play an important role in the compensatory growth of fish.

## Conclusions

In the present experiment, we carried out an analysis of the transcriptome profiles of *S. hollandi* muscle under different breeding conditions. During fasting, genes involved in fat, protein, and carbohydrate metabolism were up-regulated, which indicated that the stored energy was mobilized. On the other hand, the expression levels of genes associated with the cell cycle, DNA replication, and cellular structures decreased, which suggested that growth might be inhibited by starvation. After refeeding, most of the genes down-regulated during fasting were recovered, while many genes associated with the structure of myofibers were up-regulated. Histological analyses of muscle also showed muscle overgrowth after refeeding. The results indicated that muscle hypertrophy may play an important role in compensatory growth after fasting refeeding. The results of these experiments may help to elucidate the mechanisms underlying the starvation response and compensatory growth.

## Methods

### Sample preparation and fasting experiments

The mixed-sex, full-sib experimental fish were obtained from Shaoguan Fisheries Research Institute in Guangdong, China. A total of 210 fish with an approximate weight of 1.0 g were randomly and equally assigned to seven aquariums. As *Spinibarbus hollandi* is highly sensitive to the external stimulus, which will lead to not ingesting for several days, one aquarium was sampled only once in our experiment. Thus, among the seven aquariums, one was used for the measurements of the initial growth traits, and the remaining six aquariums were equally divided into test and control groups, corresponding to 7, 14, and 40 days. To minimize the effects introduced by different aquariums, we maintained all aquariums in the same conditions, including 100 L of charcoal-dechlorinated and continuous flow-through tap water (pH 7.0 ± 0.1) with forced-air aeration, and the temperature of the water was 26 °C with a photoperiod of 14:10 h (light:dark). The fish were manually fed twice a day to apparent satiation at 7:00 and 17:00 h, with a 35% protein/4% fat commercial diet (QiCai Pet Products Co., Ltd., Guangzhou, China) for 2 weeks. Fish served as control groups and were fed to satiation every day as mentioned above. For the test groups, fish were first deprived of food for 7 days and then fed twice per day for 33 days. For aquariums used for 7-day continuously feeding, 7-day fasting and 7-day refeeding after fasting, 15 fish were used for RNA-seq, 3 fish were used for qRT-PCR, 5 fish were used for histological analysis, and redundant 7 fish were used for prevent accidental death. For other aquariums, 3 fish were used to qRT-PCR, 5 fish were used to histological analysis, redundant fish were used to keep same breeding density in all aquariums and prevent accidental death. Eight fish were randomly selected for measure of growth traits in each aquarium. After experiments, the remained fish were bred continuously for further studies. Both groups were sampled sequentially on days 0, 7, 14, and 40. The fish were put into water with an overdose (100 ppm) of eugenol (Sangon Biotech Co., Ltd., Shanghai, China) for 10 min to anesthetize them before dissection, and their body weights, body lengths, total lengths, body thicknesses and body heights were measured. Daily gain of body weight was also calculated using the following formula:

$$ \mathrm{R}=\left(\mathrm{m}2-\mathrm{m}1\right)/\mathrm{t} $$

The R indicated the daily body weight gain ((g/day), the m1 indicated the initial weight (g), the m2 indicated the final weight after treatment (g), and t indicated the period of treatment (day).

For RNA sequencing, The muscle tissues were rapidly sampled from the same location of dorsal white muscle under dorsal fin from all fish without considering dorsal side, immediately frozen in liquid nitrogen, and stored at − 80 °C for further testing. At the same time, another three and five fish were sampled as the above methods for qRT-PCR and histology analysis, respectively. After the experiment, wherever possible, most remaining experimental fish were retained for other research.

### RNA isolation

Total RNA was extracted from approximately 80 mg of muscle tissue using RNAiso reagents (Takara, Dalian, China) according to the manufacturer’s instructions. The quantity and quality of RNA samples were determined using a microplate spectrophotometer (BioTek Company, USA) followed by electrophoresis on a 1% agarose gel. The RNA samples were stored at − 80 °C until further use.

### Library preparation and sequencing

The RNA samples prepared above were used for RNA-seq. The variation among individuals was minimized by mixing equal amounts of RNA from five samples in the same group to generate three replicated mixed RNA pools. A total of nine replicated mixed RNA samples were used for cDNA library preparation. RNA-seq libraries were prepared using NEBNext UltraTM RNA Library Prep Kit (NEB, USA) following the manufacturer’s protocols. Briefly, mRNA was purified from total RNA and was broken to fragmentations using divalent cations. First strand cDNA was synthesized using reverse transcriptase (MMLV), and second strand cDNA was synthesized using DNA polymerase I, and the remained mRNA were subsequently removed using RNase H. cDNA fragments were ligated with adaptor and then 250~300 bp fragments were selected using AMPure beads for library construction (Beckman Coulter, Beverly, USA). 3 μl USER Enzyme (NEB, USA) was added into purified cDNA fragments and incubated at 37 °C for 15 min followed by 5 min at 95 °C. cDNA fragments were amplified using polymerase chain reaction (PCR). After purified by using AMPure beads, the library quality and quantity was measured using Agilent 2100 Bioanalyzer (Agilent Technologies). Finally, the libraries were sent to Novogene Co. Ltd. to sequence using Illumina-Hiseq 2000 platform with PE-150 paired-end approach.

### RNA-seq data analyses

Raw data were filtered by removing the reads containing the adapters, poly-N, and low-quality reads, resulting in the generation of clean reads, which were assembled de novo using the Trinity software version 2.5.1 [57]. The assembled transcripts were identified using Blast+ (version: ncbi-blast-2.2.28+) against the National Center for Biotechnology Information (NCBI) nonredundant (Nr, e-value <1e-5), Clusters of Orthologous Groups of proteins (COG, e-value <1e^− 3^), Kyoto Encyclopedia of Genes and Genomes (KEGG, e-value <1e-5), and Swiss-Prot databases (e-value <1e-5). The gene ontology (GO) annotation was performed using Blast2GO with an e-value threshold of 1e-6.

The total mapped read counts for each transcript were determined using the RSEM software v1.2.15 [58]. The differential expression statistical analyses were performed using the median of ratios from the R package, DESeq [59]. The adjusted *p*-value (p-adj) with a cut-off value of 0.05 and log2 (fold change) with a cut-off absolute value of 1 were used to identify the significant differentially expressed genes (DEGs). Omicshare online website (http://www.omicshare.com/tools/Home/Soft/pathwaygsea?l=en-us) was used for Gene Ontology and KEGG pathway enrichment analysis. Significantly enriched pathways in the given gene set compared to the genome background are defined by a hypergeometric test. The formula for calculating the *P*-value is

$$ P=1-\sum \limits_{i=0}^{m-1}\frac{\left(\begin{array}{c}M\\ {}i\end{array}\right)\left(\begin{array}{c}N-M\\ {}n-i\end{array}\right)}{\left(\begin{array}{c}N\\ {}n\end{array}\right)} $$

In this equation, N is the number of all genes with KEGG pathway annotation, n is the gene number of given genes set in N, M is the number of all genes that are annotated to the certain pathway, and m is the gene number in the given gene set that is annotated to the certain pathways. The calculated p-value is gone through FDR Correction, taking FDR ≤ 0.05 as a threshold. GO terms with a q-value lower than 0.05 were further summarized with REVIGO [60]. The UpSet Chart were made by an R package UpSetR [61].

### Quantitative real-time PCR analysis

Three biological replicates were generated for each RNA sample for quantitative real-time PCR (qRT-PCR) analysis. The beta-actin gene was used as an internal control, which did not significantly change expression in the experiment (Control vs Fasting, *p* = 0.80; Control vs Refeeding, *p* = 0.82; Fasting vs Refeeding, *p* = 1.0; *exactTest,* DESeq). The primers used in the qRT-PCR analysis are shown in Additional file 1: Table S1. The qRT-PCR analysis was performed using the ABI 7000 platform in 20 μL reactions containing the following components: 100 ng of cDNA, 10 μL Power SYBR Green PCR Master Mix (Vazyme Biotech, Nanjing, China), 0.3 μL of each primer (10 μM), and 7.4 μL double-distilled water. The reaction procedure was as mentioned in the manufacturer’s instructions for the SYBR Green PCR Master Mix. All samples were analyzed in triplicate, and fold changes were calculated using the comparative Ct method (also known as the 2^−ΔΔCt^ method). All data are provided in terms of the relative mRNA expression as the mean ± S.E. (*n* = 3).

### Muscle histology

For muscle morphology observation, five fish from each group were selected. The dorsal muscle tissues obtained in the preceding experiment were fixed for 24 h in Bouin solution. The tissues were then dehydrated using an ethyl alcohol series, hyalinized in xylene baths, and embedded in paraffin. After the paraffin block solidified, thin sections (6 μm) were cut using a rotary microtome (Cut 4055; Olympus American, Melville, NY, USA). The sections were flattened, stained with hematoxylin and eosin, mounted on slides with neutral resin, and examined using light microscopy. The longest and shortest diameters of myofibers were measured and recorded for the control and test groups. A total of ~ 100 cells of each group were randomly selected for measurement. For each fish, a clear vision was used to measure cell size, and approximately 20 cells of each vision were randomly measured and recorded.

### Statistical analyses

All data were analyzed using SPSS Statistics software version 20 (IBM, Chicago, IL, USA) and presented as the means ± SEM. The body weight and myocyte sizes of fish in the control and test groups were analyzed using *Student’s t-test*. *P*-values < 0.05 were considered to be statistically significant.

## Supplementary information

**Additional file 1: Table S1.** Primer pairs used for qRT-PCR. **Figure S1.** Daily changes of growth traits. **Figure S2.** The expression of genes associated with structure of myofiber.

**Additional file 2: Table S2. Figure S2.** The list of all differentially expressed genes during fasting-refeeding.

## Data Availability

All the data obtained in the current study have been presented in this article. The RNA-Seq sequence raw data-set supporting the results of this study have been deposited at the National Center for Biotechnology Information (NCBI), sequence read archive (SRA) database, and the accession number is SRP133262.

## Abbreviations

COG: Clusters of Orthologous Groups of proteins
DEGs: Differentially expressed genes
FPKM: Fragment Per Kilobase of transcript sequence per Millions
GO: Gene ontology
HSPs: Heat shock proteins
KEGG: Kyoto Encyclopedia of Genes and Genomes
MCM: Mini-chromosome maintenance
NCBI: National Center for Biotechnology Information ()
Nr: Non-redundant protein (NR), NCBI database
Nt: Non-redundant nucleotide (Nt), NCBI database
qRT-PCR: Quantitative real-time PCR
