# Supplementary material for: Transcriptional profiling of skeletal muscle reveals starvation response and compensatory growth in Spinibarbus hollandi
Source: BMC Genomics. 2019 Dec 5;20:938. doi: 10.1186/s12864-019-6345-2 (PMC6896686; doi:10.1186/s12864-019-6345-2)
Supplement: Supplementary file 1 — Additional file 1: Table S1. Primer pairs used for qRT-PCR. Figure S1. Daily changes of growth traits. Figure S2. The expression of genes associated with structure of myofiber. [file 12864_2019_6345_MOESM1_ESM.docx]

**Supplementary Information of “Transcriptional profiling of skeletal muscle reveals starvation response and compensatory growth in *Spinibarbus hollandi*”**

**Supplementary Information includes:**

Supplementary Table 1

Legend for Supplementary Figure 1~ 2

Supplementary Figure 1~ 2

Table S1 Primer pairs used for qRT-PCR

| **Genes** | **Production Length (nt)** | **Primers（5′-3′）** | **Length (nt)** | **Purpose** |
| --- | --- | --- | --- | --- |
| ACT2F | 106 | GCCTGCCTCATCATACTCCTGCTT | 24 | qRT-PCR |
| ACT2R | 106 | CCCACCTGAGCGTAAATACTCCGT | 24 | qRT-PCR |
| ACTA1F | 121 | GCGACATTGACATCAGGAAGGACC | 24 | qRT-PCR |
| ACTA1R | 121 | CACGACGCTCTTCCGATCTCAGT | 23 | qRT-PCR |
| CDK1F | 102 | TCGATGCCGTTCTTGTCCAGGTT | 23 | qRT-PCR |
| CDK1R | 102 | GCCAGATGTTGAGTCGCTACCAGA | 24 | qRT-PCR |
| MCHIIF | 110 | AGATCATGCCAGCTCCACTGAACC | 24 | qRT-PCR |
| MCHIIR | 110 | TGATTCCCAGCACCAGACCAGATG | 24 | qRT-PCR |
| RNF25F | 115 | AGTCACTCCCTTCCTCGTCCCAA | 23 | qRT-PCR |
| RNF25R | 115 | TCACCTTGCTGCCTTCGCTCAT | 22 | qRT-PCR |
| SLC27A3F | 109 | CCTGACCAGCTCCGTCTATGACAG | 24 | qRT-PCR |
| SLC27A3R | 109 | TGTCGCTCATCGTCTGCTGCTAA | 23 | qRT-PCR |
| MCM4F | 105 | AGGAGAGGAGGGCTCGGATAGAAC | 24 | qRT-PCR |
| MCM4R | 105 | CAAGGTGGAGGTTGTGGCTGAGA | 23 | qRT-PCR |
| TAP1F | 104 | CAGCATGGAGCCGAACAGGAAGA | 23 | qRT-PCR |
| TAP1R | 104 | GCATCACCACCGACACCAACAC | 22 | qRT-PCR |
| TMSBF | 124 | ACTCTCCTCGCCTCTCCTGTTCA | 23 | qRT-PCR |
| TMSBR | 124 | GCTGACAAACCCAACATGACGGAA | 24 | qRT-PCR |
| TNNT3F | 96 | CTACAGGCCATCAGCACCCAAGA | 23 | qRT-PCR |
| TNNT3R | 96 | TGCAGCTCAAGAGTATCCTTGTTCTG | 26 | qRT-PCR |
| BETAACTINF | 109 | AGAGGACGCAGTCAAAGCTCAGAG | 24 | internal control |
| BETAACTINR | 109 | ACCATGTACCCTGGCATTGCTGAT | 24 | internal control |


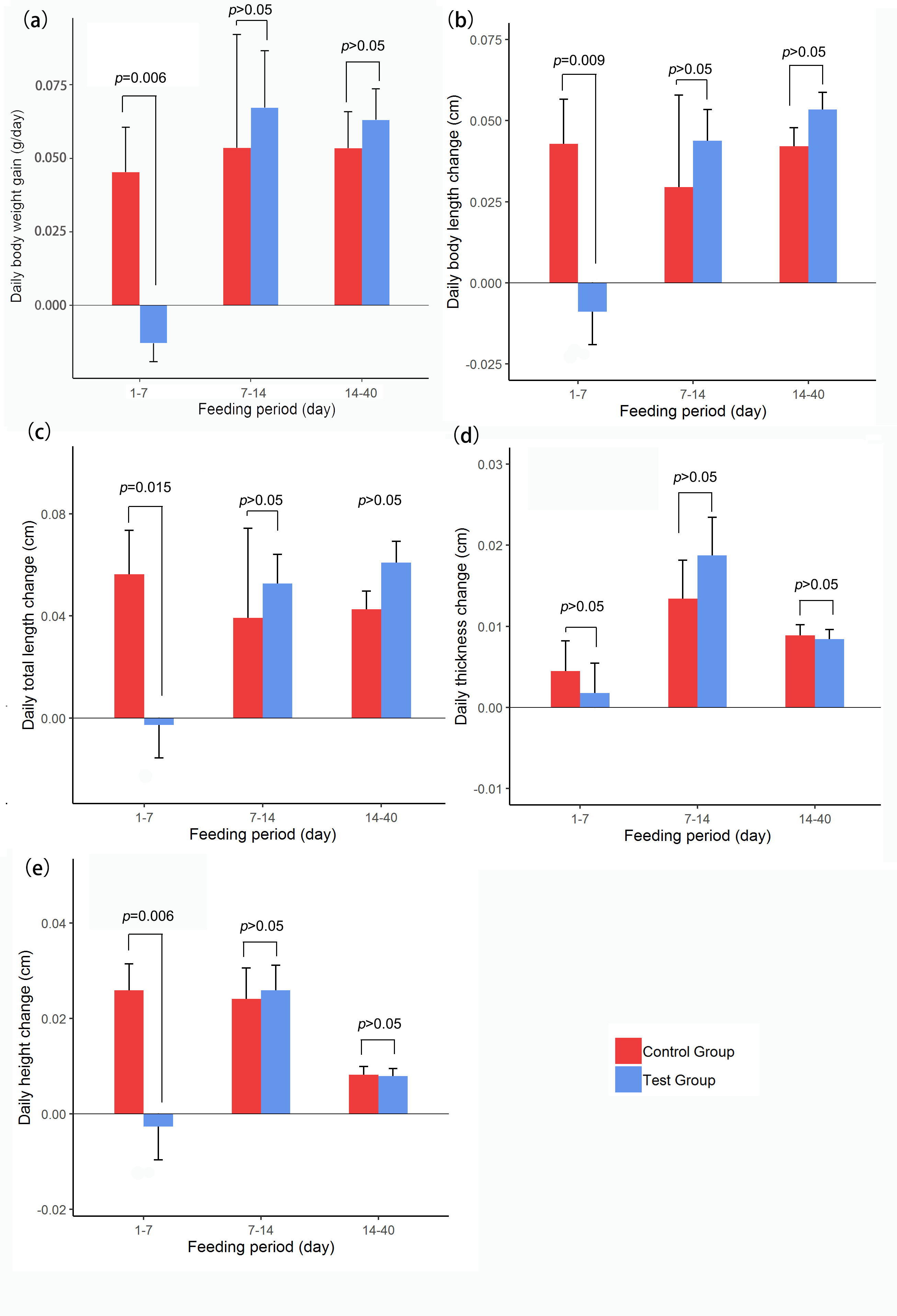


**Figure S1 Daily changes of growth traits**. **a)** Body weight **b)** Body length; **c)** Total length; **d)** Thickness; **e)** height

Asterisks indicate significant difference between the control and test groups (*Student’s t-test; df*=9)


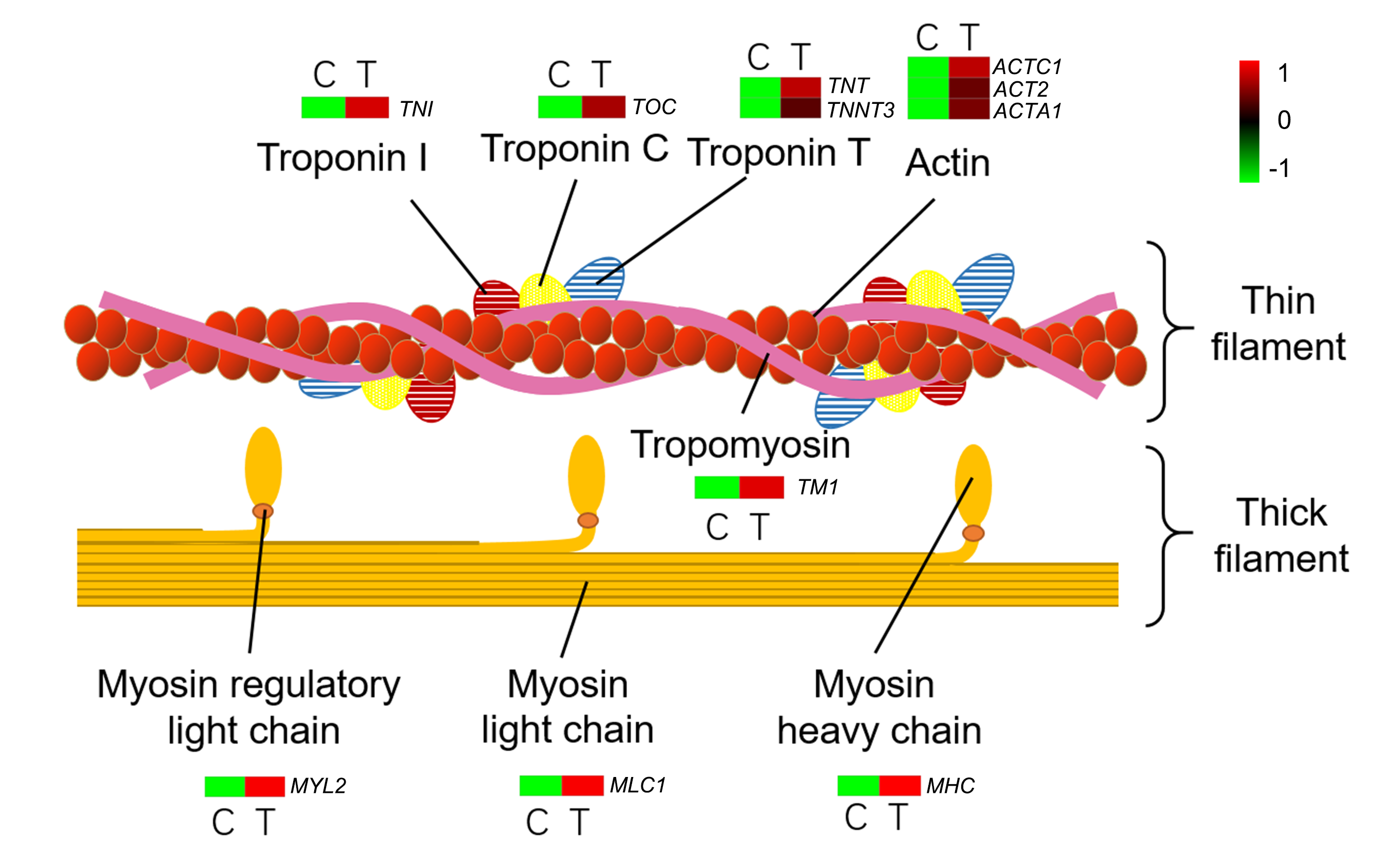


**Figure S2 The expression of genes associated with structure of myofiber.** C indicated control group; T indicated test group (after 7 days refeeding), the lighter red indicated the higher expression level.
